# GDF-15 as a Therapeutic Target of Diabetic Complications Increases the Risk of Gallstone Disease: Mendelian Randomization and Polygenic Risk Score Analysis

**DOI:** 10.3389/fgene.2022.814457

**Published:** 2022-06-13

**Authors:** Lili Yu, Yajing Zhou, Lijuan Wang, Xuan Zhou, Jing Sun, Jiarui Xiao, Xiaolin Xu, Susanna C. Larsson, Shuai Yuan, Xue Li

**Affiliations:** ^1^ Department of Big Data in Health Science School of Public Health, Center of Clinical Big Data and Analytics of The Second Affiliated Hospital, Zhejiang University School of Medicine, Hangzhou, China; ^2^ Unit of Cardiovascular and Nutritional Epidemiology, Institute of Environmental Medicine, Karolinska Institutet, Stockholm, Sweden; ^3^ Unit of Medical Epidemiology, Department of Surgical Sciences, Uppsala University, Uppsala, Sweden; ^4^ Centre for Global Health Research, Usher Institute, University of Edinburgh, Edinburgh, United Kingdom

**Keywords:** gallstones, growth differentiation factor 15, metformin, Mendelian randomization, diabetic complication

## Abstract

Growth differentiation factor 15 (GDF-15) levels have been revealed as a robust biomarker for metformin use. We conducted Mendelian randomization (MR) analysis to explore the association between GDF-15 and gallstone disease to inform potential therapeutic effects targeting GDF-15. Four genetic variants associated with GDF-15 levels at *p* < 5 × 10^–8^ were selected as instrumental variables from a genome-wide association meta-analysis including 21,758 individuals. Two-sample MR analysis was conducted using summary-level data from UK Biobank (10,520 gallstone cases and 350,674 controls) and FinnGen consortium (19,023 gallstone cases and 195,144 controls). Polygenic risk score analysis using individual-level data in UK biobank was performed to complement the MR findings by examining the non-linearity of the association. Diabetic complications were taken as positive controls to validate the therapeutic effect of targeting GDF-15. Linear and nonlinear associations between genetically predicted GDF-15 levels and gallstones were estimated with stratification by the diabetic status. In the two-sample MR analysis, the odds ratio (OR) of gallstones was 1.09 (95% confidence interval (CI), 1.03–1.15; *p* = 0.001) for one standard deviation increase in genetically predicted GDF-15 levels in the meta-analysis of two datasets. Polygenic risk score analysis found this association to be U-shaped (*p* = 0.037). The observed association was predominantly seen in nondiabetic population (OR = 1.11, 95% CI: 1.01–1.21; *p* = 0.003). An inverse association between genetically predicted GDF-15 levels and diabetic complications (OR = 0.77, 95% CI: 0.62–0.96; *p* = 0.023) was observed, validating the potential therapeutic effects of targeting GDF-15 levels. This MR study indicates that the increased risk of gallstone disease should be taken into account when considering GDF-15 as a therapeutic target for diabetic complications.

## Introduction

Growth differentiation factor 15 (GDF-15) is one of the members of transforming growth factor beta (TGF-β), specifically produced by macrophages and epithelial cells, as well as by adipocytes, with the basic effects on inhibition of macrophage activation, anti-inflammation, and maintenance of homeostasis (Bootcov et al., 1997; Unsicker et al., 2013).

Experimental data on mice found that GDF-15 controlled appetite (Johnen et al., 2007; Macia et al., 2012), reduced body weight and fat mass (Johnen et al., 2007; Macia et al., 2012; Chrysovergis et al., 2014), increased metabolism rate (Chrysovergis et al., 2014), and improved glycemic profiles (Li et al., 2013). A high expression of GDF-15 has been proposed to be a predicting factor (Bao et al., 2019) and a potential treatment target for diabetes mellitus along with complications (Nakayasu et al., 2020; Xiao et al., 2022).

Evidence from some population-based studies has also been provided that increased GDF-15 might be a cause mediator or predicting biomarker in health-adverse outcomes, such as insulin resistance (Vila et al., 2011; Kempf et al., 2012) and cardiovascular disease (Brown et al., 2002; Wollert et al., 2017). Recently, GDF-15 levels have been revealed as a robust biomarker for metformin use (Gerstein et al., 2017; Day et al., 2019), which is well-recognized as the first-line medication for diabetes (Suissa and Azoulay, 2012; Griffin et al., 2017). The medication can lower diabetic complications and has been suggested to have potential benefits in cardiovascular disease and cancer prevention in diabetic individuals, although corresponding evidence remains inconclusive. However, its secondary effects on gastrointestinal complaints, including gallstones, are scarcely evaluated due to lack of high-quality data (Schlender et al., 2017).

Gallstone disease is defined by the occurrence of symptoms or complications caused by gallstones in the gallbladder and/or the bile ducts (Lammert et al., 2016), which has been proposed to be a potential secondary disease of diabetes (Lieber, 1952; Everhart and Ruhl, 2009), obesity (Yuan et al., 2021), and insulin resistance (Nervi et al., 2006), possibly through increased hepatic cholesterol uptake and synthesis, gallbladder hypomotility, etc. Of note, some studies have observed a higher expression of GDF-15 in patients with benign biliary diseases like gallstone disease, cholecystitis, etc. than in healthy controls (Li et al., 2020; Ozkan et al., 2011), providing a new perspective for exploration of the novel role of GDF-15 in the progress of gallstone disease. In spite of limited direct evidence on GDF-15 in relation to gallstones, the associations of GDF-15 or gallstones, respectively, with diabetes indirectly support and enrich the hypothesis.

Employing genetic variants as instrumental variables for exposure (e.g., GDF-15 levels) and Mendelian randomization (MR) analysis can strengthen causal inference by minimizing unobserved confounding and diminishing reverse causality (Burgess and Thompson, 2015). Therefore, we conducted a two-sample Mendelian randomization (MR) study and polygenic risk score analysis, here, to determine the association between genetically predicted GDF-15 levels, a biomarker of metformin use, and the risk of gallstones.

## Methods

### Study Design and Data Sources

**FIGURE 1:**
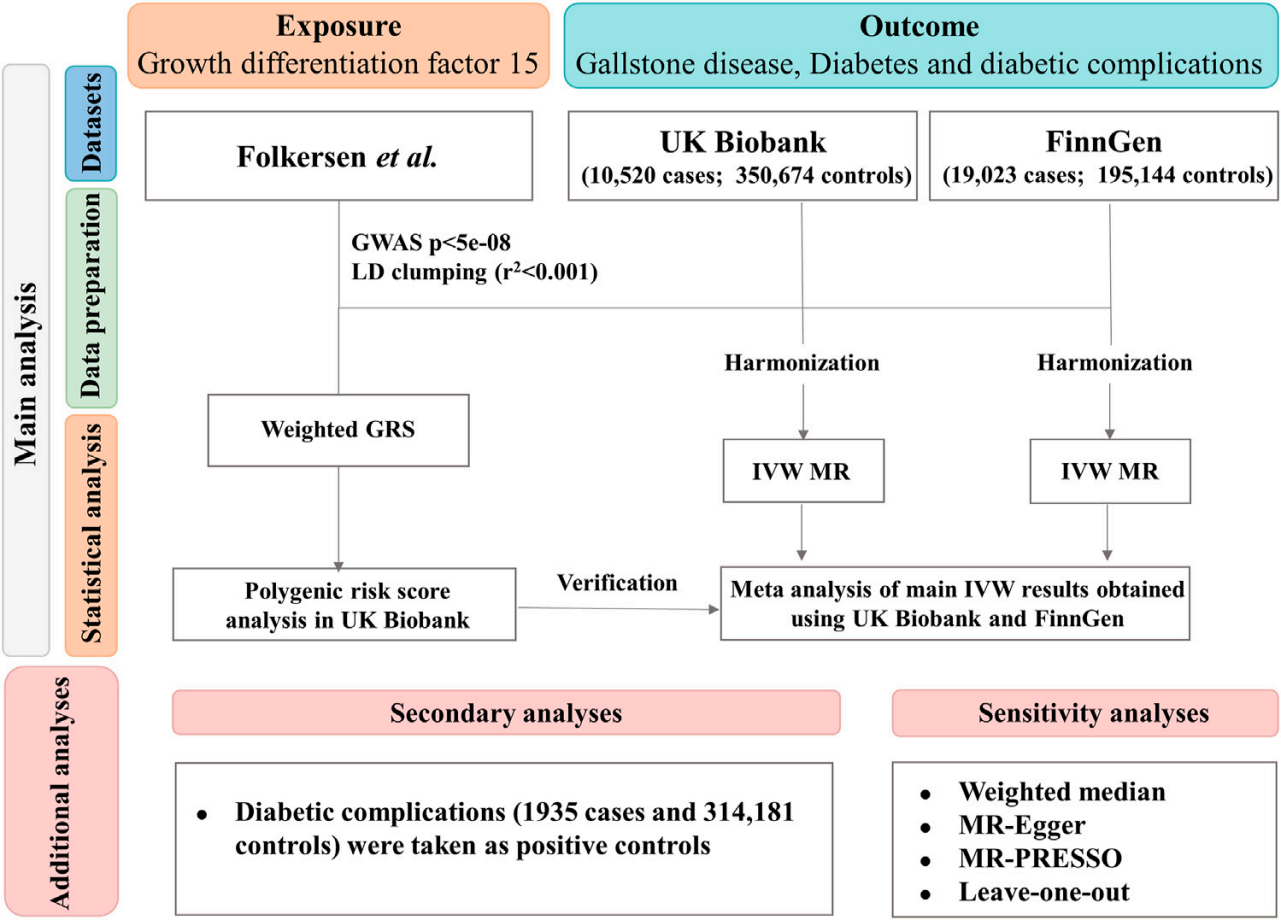
Schematic representation of the study design. MR, Mendelian randomization; IVW, inverse variance weighted; GRS, weighted genetic risk score; LD, linkage disequilibrium; GWAS, genome-wide association study.

Figure 1 shows the overview of the study design. We first performed a two-sample MR study based on the UK Biobank study and the FinnGen study to determine the causal association between genetically predicted GDF-15 and gallstone disease. We then conducted a polygenic risk score analysis based on individual-level data from the UK Biobank study to further explore the diabetes-specific association and the nonlinearity of the association. UK Biobank received ethical approval from the North West Multi-centre Research Ethics Committee, the National Information Governance Board for Health and Social Care in England and Wales, and the Community Health Index Advisory Group in Scotland. All participants provided written informed consent. The ethical permit for MR analyses based on summary-level data was unnecessary.

### Genetic Instrument Selection

Genetic variants associated with GDF-15 levels at the genome-wide significance threshold (*p* < 5 × 10^–8^) were identified from a meta-analysis of 13 genome-wide association studies including up to 21,758 individuals of the European ancestry (Folkersen et al., 2020). Linkage disequilibrium was calculated based on the 1,000 genomes European reference panel (Clarke et al., 2012). Four single nucleotide polymorphisms (rs2517481, rs1227734, rs60164552, and rs112253475) without linkage disequilibrium (*r* ^*2*^ ≤ 0.01 and clump window > 10,000 kb) were selected as instrumental variables for GDF-15 (Table 1). These SNPs explain approximately 8.4% of phenotypic variance. The association test was adjusted for population structure and study-specific parameters (Folkersen et al., 2020). Standard sample-level quality control (QC) exclusions were based on call rate filters, sex mismatch, population outliers, heterozygosity, and cryptic relatedness. (Folkersen et al., 2020).

**TABLE 1:** Genetic instruments for growth differentiation factor 15 levels and their associations with gallstones.

SNP	Chr	Position	Gene	EA	NEA	EAF	GDF-15			Gallstones in UKB			Gallstones in FinnGen		
							Beta	SE	*p-*value	Beta	SE	*p-*value	Beta	SE	*p-*value
rs2517481	6	31043931	*TBC1D22B*	G	C	0.59	0.059	0.010	1.7e-08	0.001	0.014	0.975	0.001	0.014	0.971
rs1227734	19	18501034	*GDF15*	T	C	0.14	0.370	0.013	9.9e-177	0.066	0.020	6.8e-4	0.025	0.023	0.281
rs60164552	19	18488285	*GDF15*	G	C	0.92	0.160	0.017	7.9e-22	−0.026	0.027	0.337	0.032	0.024	0.175
rs112253475^a^	19	18841757	*CRTC1*	A	G	0.98	0.270	0.044	6.5e-10	−0.015	0.062	0.806	0.006	0.038	0.871

Chr, chromosome; EA, effect allele; EAF, effect allele frequency; NEA, non-effect allele; SE, standard error; SNP, single nucleotide polymorphism; UKB, UK Biobank. The position was based on human genome 19 (hg19).

a Rs112253475 was unavailable in FinnGen and proxied by rs118170439 (*r* ^2^ = 0.8).

### Two-Sample MR Analysis

Two-sample MR analyses were performed using summary-level data for the association of GDF-15–associated SNPs with gallstone disease from the UK Biobank study (10,520 cases and 350,674 controls) and the FinnGen study (19,023 cases and 195,144 controls). We used the Neale Lab second wave results of the genome-wide analysis in UK Biobank after the removal of individuals of the non-European ancestry, closely related individuals, individuals with sex chromosome aneuploidies, and individuals who had withdrawn consent. The association test was adjusted for age, sex, and up to 20 genetic principal components. The R5 release of FinnGen consortium data was used, where individuals with ambiguous gender, high genotype missingness, excess heterozygosity, and non-Finnish ancestry had been excluded. The association test in FinnGen was adjusted for age, sex, 10 genetic principal components, and genotyping batch.

### Polygenic Risk Score Analysis

Polygenic risk score analysis was conducted in the UK Biobank study including 16,463 gallstone patients and 318,308 controls after exclusion of individuals with high missingness or heterozygosity, outlying short runs of homozygosity, sex mismatch, and non-British ancestry (to minimize population bias). Incident and prevalent gallstone cases were defined by corresponding codes in the International Classification of Diseases (ICD) versions 9 and 10 with information from national medical records (including inpatient hospital episode records, primary general practitioner data, cancer registry, and death registry). Diabetic complications, including type 2 diabetes with ketoacidosis, renal manifestations, ophthalmic manifestations, and neurological manifestations (1,935 cases and 314,181 controls) were taken as positive controls to validate the effect of GDF-15 as a therapeutic target for diabetic complications. Individual-level UK Biobank data was accessed up to 6^th^ March 2021 under the approved project application (ID: 66345). Polygenic risk scores for GDF-15 levels were constructed by summing the number of risk alleles (each SNP was recoded as 0, 1, or 2) carried by each participant and weighted by the effect size estimates (i.e., beta coefficient) of the four genetic instrument variables (Table 1) selected for GDF-15.

### Statistical Analysis

For two-sample MR analysis, the inverse-variance weighted method with random effects was used as the main method and supplemented by four sensitivity analyses, including the weighted median (Bowden et al., 2016), MR-Egger regression (Bowden et al., 2015), MR-PRESSO (Verbanck et al., 2018), and leave-one-out analysis. The weighted median method can provide consistent causal estimates if more than 50% of the weight comes from valid instrumental variables (Bowden et al., 2016). MR-Egger can generate estimates after correcting for horizontal pleiotropy; however, this method compromises statistical power (Bowden et al., 2015). MR-PRESSO can detect outlying instrumental variables and provide causal estimates after the removal of these outliers (Verbanck et al., 2018). The leave-one-out analysis can detect whether the association is driven by a certain SNP. In addition, we made a scatterplot to show the association for each SNP. Cochrane’s Q value was used to assess the heterogeneity among estimates of genetic instruments, and the *p*-value for the intercept in MR-Egger was used to detect horizontal pleiotropy (Bowden et al., 2015). Possible pleiotropic associations with GDF-15-associated SNPs were searched in PhenoScanner V2, a database of human genotype–phenotype associations (Kamat et al., 2019).

For polygenic risk score analysis, logistic regression was first used to explore the linear association between the polygenic score of GDF-15 and gallstone risk. We have three levels of models: 1) the univariate regression model without adjustment for any covariates; 2) the multivariable regression model with adjustment for basic covariates, including age, sex, body mass index (BMI), and the first 20 genetic principal components; and 3) the multivariable regression model with adjustment for a comprehensive list of covariates, including age, sex, BMI and the first 20 genetic principal components, deprivation index, smoking, drinking, physical activity, blood low-density lipoprotein cholesterol (LDL-C) levels, blood glucose levels, blood HbA1c levels, and metformin use. Considering confounding effects from type 2 diabetes (Yuan et al., 2021) and most diabetic patients had taken metformin, in whom the genetically predicted GDF-15 cannot proxy the true level of exposure, we stratified the association by diabetic status. We also examined the nonlinearity of the association by using logistic regression with restricted cubic splines. Spline knot from nonlinearity regression was used as a cutoff to stratify the study population into genetically predicted low and high GDF-15 groups, and the linear association between the polygenic score of GDF-15 and gallstone risk was assessed within each group separately. The nonlinearity regression of polygenic score analysis was conducted using the rms package, and MR analyses were performed using the TwoSampleMR and MR-PRESSO packages (Hemani et al., 2018; Verbanck et al., 2018) in R Software 4.0.2.

## Results

### Two-Sample MR Analysis

**FIGURE 2:**
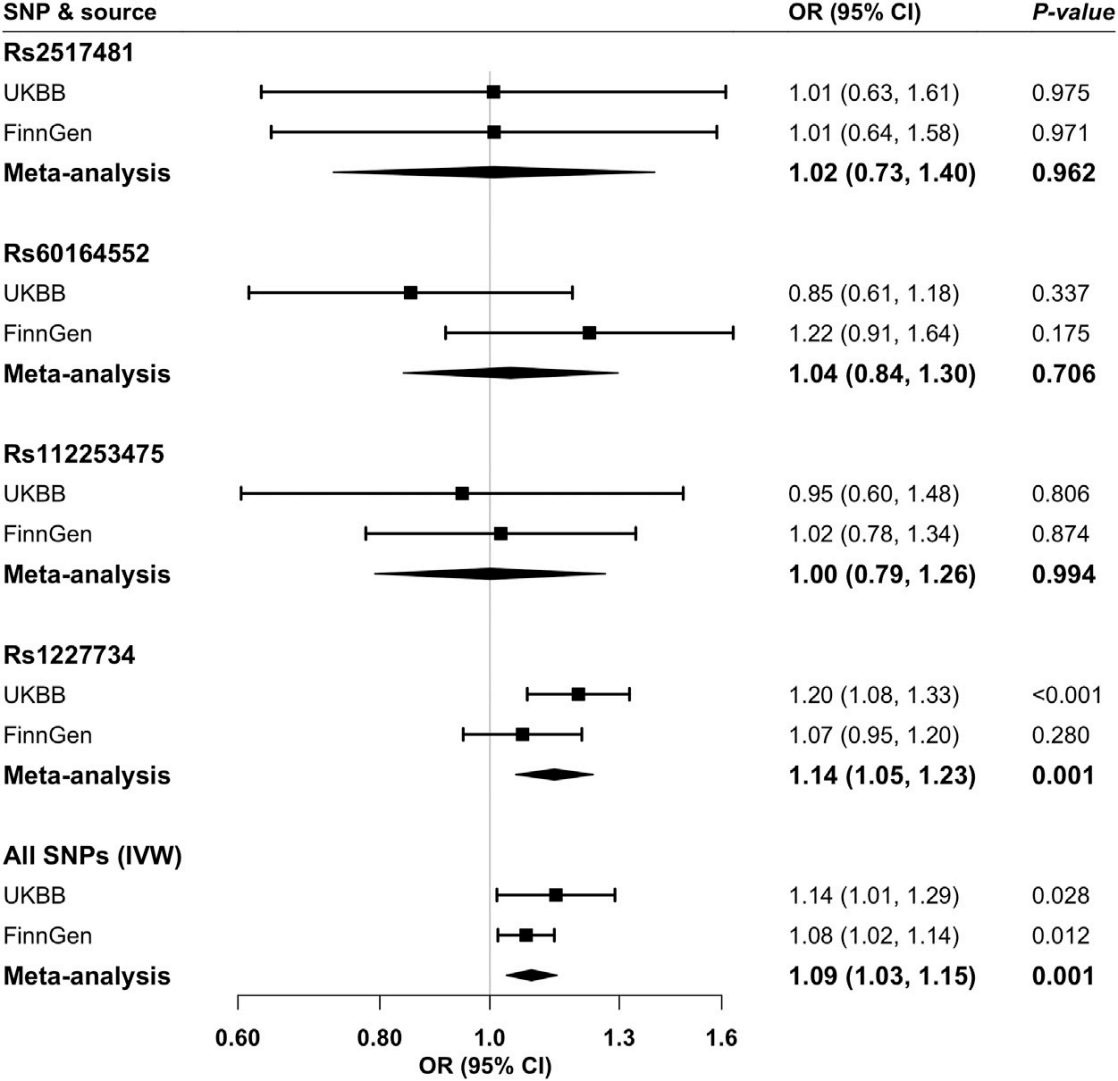
Association of genetically predicted growth differentiation factor 15 levels with gallstone disease risk in Mendelian randomization analysis. CI, confidence interval; IVW, inverse variance weighted; OR, odds ratio; SNPs, single nucleotide polymorphisms; UKBB, UK Biobank. Estimates of analyses based on all SNPs were derived from the random-effects inverse-variance weighted method.

Higher genetically predicted GDF-15 levels were associated with an increased risk of gallstone disease (Figure 2). For one standard deviation increase in genetically predicted GDF-15 levels, the odds ratio (OR) of gallstones was 1.14 (95% confidence interval [95% CI]: 1.01, 1.29; *p* = 0.028) in UK Biobank, 1.08 (95% CI: 1.02, 1.14; *p* = 0.012) in FinnGen, and 1.09 (95% CI: 1.03, 1.15; *p* = 0.001) in the meta-analysis of the two datasets. Four GDF-15–associated SNPs showed consistent associations with gallstones in FinnGen (Supplementary Figure S1). The association was driven by rs1227734 which explains the majority of explained variance of GDF-15 (Figure 2; Supplementary Figure S2). The association was consistent in sensitivity analyses (Table 2). We detected moderate heterogeneity between estimates of SNPs, no indication of horizontal pleiotropy in MR-Egger (*p* for intercept >0.05), and no outlier in MR-PRESSO analysis (Table 2). Rs2517481 but no other SNPs showed possible pleiotropic associations with other phenotypes (Supplementary Table S1).

**TABLE 2:** Association of genetically predicted growth differentiation factor 15 levels with gallstone disease risk in Mendelian randomization sensitivity analysis.

Source	Method	OR	95% CI	*p-*value
UK Biobank	Weighted median	1.16	1.05, 1.28	0.005
	MR-Egger	1.23	1.01, 1.49	0.176
	Cochrane’s Q = 0.97 (*p* = 0.808); MR-Egger intercept = 0.000 (*p* = 0.994)			
FinnGen	IVW-random effects	1.06	0.95, 1.19	0.261
	Weighted median	1.08	0.92, 1.26	0.467
	Cochrane’s Q = 4.81 (*p* = 0.186); MR-Egger intercept = −0.020 (*p* = 0.454)			

CI, confidence interval; NA, not available; OR, odds ratio. No outlier was detected in MR-PRESSO, analysis in UK Biobank, or FinnGen, and so the estimates from MR-PRESSO were identical to those from the main analysis.

### Polygenic Risk Score Analysis

**FIGURE 3:**
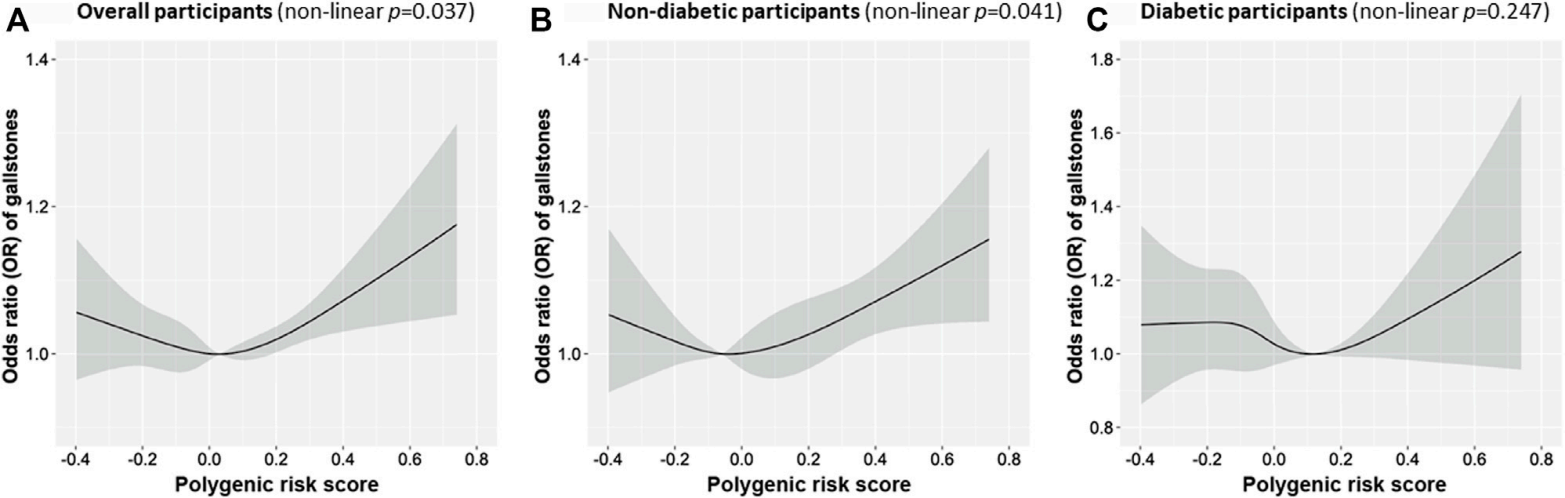
Nonlinear regression of association between the polygenic risk scores of growth differentiation factor 15 and gallstones in overall participants **(A)**, nondiabetic participants **(B)** and diabetic participants **(C)**. Associations were adjusted for a comprehensive list of covariates, including age, sex, BMI and the first 20 genetic principal components, deprivation index, smoking, drinking, physical activity, blood LDL-c levels, blood glucose levels, blood HbA1c levels, and metformin intake.

Characteristics of 339,197 participants in the UK Biobank are shown in Table 3. The population with gallstones was more likely to be females with higher body mass index, diabetes, and metformin use. A positive association between genetically predicted GDF-15 levels and gallstone disease was observed in the polygenic risk score analysis (Table 4). The OR of gallstones was 1.11 (95% CI: 1.03, 1.19; *p* = 0.008) for one standard deviation increase in genetically predicted GDF-15 levels in a univariate model, and the effect estimate remained significant (OR = 1.10, 95% CI: 1.03, 1.19; *p* = 0.027) after adjustment for additional full list of covariates. In the stratification analysis by diabetic status, the association between genetically predicted GDF-15 and gallstones was consistently observed in nondiabetic participants (OR = 1.11, 95% CI: 1.01, 1.21; *p* = 0.003), indicating the observed association was not driven by diabetes (Table 4). In diabetic individuals, the association was insignificant (OR = 1.06, 95% CI: 0.85, 1.32; *p* = 0.605), and the power calculation indicated insufficient statistical power (13%) to detect this effect among diabetic individuals. Furthermore, we found a nonlinear U-shaped association by a regression model with cubic splines (the turning point of polygenic risk score of -0.06, *p* for nonlinearity = 0.037) (Figure 3). When restricting to nondiabetic individuals, genetically predicted GDF-15 levels showed an inverse but non-significant association with gallstone risk (OR = 0.82, 95% CI: 0.59, 1.13; *p* = 0.228) when it is below the turning point, while genetically predicted high levels of GDF-15 (above the turning point) showed a significant positive association with an increased risk of gallstones (OR = 1.28, 95% CI: 1.90, 1.43; *p* = 0.002).

**TABLE 3:** Characteristics of participants in the UK Biobank.

Characteristic	With gallstones	All participants
	Mean (SD)/N (%)	Mean (SD)/N (%)
N	16,463	339,197
Age, mean (SD)	58.6 (7.6)	56.9 (8.0)
Female, N (%)	11,065 (67.2)	182,072 (53.7)
BMI, kg/m^2^, mean (SD)	29.72 (5.4)	27.4 (4.8)
SBP, mmHg, mean (SD)	141.0 (19.4)	139.0 (18.7)
On antihypertensive medications, N (%)	3,704 (22.5)	74,507 (22.0)
LDL cholesterol, mmol/L, mean (SD)	3.54 (0.9)	3.57 (0.9)
On lipid-lowering medications, N (%)	3,202 (19.4)	63,652 (18.8)
HbA1c, mmol/mol, mean (SD)	37.09 (7.9)	35.95 (6.5)
History of diabetes mellitus, N (%)	2,573 (15.6)	26,100 (7.7)
On metformin medications, N (%)	677 (4.1)	8,207 (2.4)
Current smoker, N (%)	1725 (10.5)	34,024 (10.0)

BMI: body mass index; HbA1c: glycated hemoglobin A1c; LDL: low-density lipoprotein; SBP: systolic blood pressure.

**TABLE 4:** Association of genetically predicted growth differentiation factor 15 levels with gallstone disease risk in UK Biobank.

	Overall participants (N = 339,197)		Nondiabetic participants (N = 313,097)		Diabetic participants (N = 26,100)	
	OR (95%CI)	*p-*value	OR (95%CI)	*p-*value	OR (95%CI)	*p-*value
**Overall**						
Model 1	1.11 (1.03, 1.19)	0.008	1.12 (1.03, 1.22)	0.005	1.04 (0.85, 1.25)	0.735
Model 2	1.11 (1.03, 1.19)	0.009	1.12 (1.03, 1.21)	0.009	1.05 (0.86, 1.28)	0.617
Model 3	1.10 (1.01, 1.19)	0.027	1.11 (1.01, 1.21)	0.003	1.06 (0.85, 1.32)	0.605
**Genetically predicted low GDF-15 group**						
Model 1	0.85 (0.69, 1.06)	0.148	0.91 (0.68, 1.22)	0.525	0.98 (0.49, 1.98)	0.970
Model 2	0.83 (0.67, 1.03)	0.084	0.88 (0.65, 1.18)	0.393	0.96 (0.47, 1.96)	0.919
Model 3	0.78 (0.61, 0.99)	0.039	0.82 (0.59, 1.13)	0.228	0.84 (0.38, 1.85)	0.667
**Genetically predicted high GDF-15 group**						
Model 1	1.16 (0.92, 1.47)	0.218	1.24 (1.09, 1.40)	8.55 × 10^–4^	1.18 (0.87, 1.59)	0.282
Model 2	1.14 (0.90, 1.45)	0.286	1.24 (1.09, 1.41)	9.04 × 10^–4^	1.24 (0.91, 1.68)	0.169
Model 3	1.09 (0.83, 1.42)	0.540	1.28 (1.90, 1.43)	0.002	1.18 (0.84, 1.64)	0.330

CI, confidence interval; OR, odds ratio.

Model 1: univariate regression model without adjustment for any covariates; Model 2: multivariable regression model with adjustment for basic covariates, including age, sex, BMI, and the first 20 genetic principal components; Model 3: multivariable regression model with adjustment for a comprehensive list of covariates, including age, sex, BMI and the first 20 genetic principal components, deprivation index, smoking, drinking, physical activity, blood LDL-c, levels, blood glucose levels, blood HbA1c levels, and metformin intake.

### Secondary Analysis of Positive Controls

Higher genetically predicted GDF-15 levels were associated with a decreased risk of diabetic complications, validating the therapeutic effect on diabetic complications targeting GDF-15 levels; the corresponding OR was 0.77 (95% CI: 0.62–0.96; *p* = 0.023) for one standard deviation increase in genetically predicted GDF-15 levels.

## Discussion

In this first study on GDF-15 in relation to gallstones, we found an association between genetically predicted GDF-15 levels and the risk of gallstone disease. This association was confined to nondiabetic individuals and appeared to be U-shaped even though a significant association was observed for high genetically predicted GDF-15 levels but not for low genetically predicted GDF-15 levels. We observed an inverse association between GDF-15 levels and diabetic complications, which strengthened the potential usage of GDF-15 levels as a therapeutic target for diabetic complications. Taken together, our findings implied a possible adverse outcome, gallstones, when considering GDF-15 as a potential therapeutic target for diabetic complications.

Our study found that genetically predicted GDF-15 levels were significantly associated with gallstones, which support our hypothesis. Also, evidence from previous studies has explored the association between GDF-15 and gallstone disease. A case-control study observed a higher level of GDF-15 in patients with benign biliary diseases (acute cholecystitis, acute cholangitis, choledocholithiasis, and cholelithiasis), suggesting GDF-15 to be a potential biomarker in biliary diseases (Ozkan et al., 2011). Likewise, another case-control study found that the levels of GDF-15 were significantly higher in non-cirrhotic primary biliary cholangitis (PBC) patients than in healthy controls, along with a higher expression of total bilirubin (TBIL) and direct bilirubin (DBIL) (Li et al., 2020), the typical markers of cholestasis, showing the notable role of GDF-15 as a risk predictor for cholestasis and cholelithiasis.

There were also some studies indicating opposite findings to ours. An experiment in mice found that metformin that elevates circulating GDF-15 levels (Coll et al., 2020) reduced the risk of gallstones induced by a high-fat diet (Dorvash et al., 2018). In a population-based study involving 36 patients with polycystic ovary syndrome and 20 healthy controls, metformin treatment was associated with an improved metabolic and hormonal imbalance associated with polycystic ovary syndrome and increased gallbladder motility (Isik et al., 2012) that is associated with a lower risk of cholesterol gallstones (Pauletzki et al., 1996). A cohort study in diabetes also observed a reduced risk of gallstones in individuals with >180 cumulative defined daily dose of metformin compared to those with <29 cumulative defined daily dose of metformin (Liao et al., 2017). Even though these findings nominally are conflicting with our findings, several issues in previous studies deserve consideration, including lack of direct GDF-15 measurement, unadjusted important confounders (e.g., smoking status), no examination of the nonlinearity of the association, and certain special features of the diabetic population and patients with polycystic ovary syndrome. In addition, time-related biases, such as immortal time bias, time-window bias, and time-lag bias, have been acknowledged as predominant methodological issues in observational studies on metformin use (Suissa and Azoulay, 2012), which may mislead the association between metformin use and gallstones.

In stratification analysis, the significant association between higher genetically predicted GDF-15 and gallstones was only observed in nondiabetic individuals. The insignificant association in diabetic patients could be due to the lack of study power (13%) and the possibility that genetic polymorphisms may explain a smaller variance of GDF-levels, given that metformin treatment is much more frequent in diabetic patients. In addition, we observed a U-shaped association between GDF-15 and gallstone disease. The nonlinearity of the observed association has important clinical implications on the dose of metformin use which should maintain GDF-15 at a reasonable level to balance the therapeutic effects on diabetic complications and the risk of gallstone disease.

Even though the underlying mechanisms that explain the positive association between GDF-15 and gallstone disease have not been expounded, several possible pathways have been proposed. Increased GDF-15 levels are mostly linked to inflammation (Wischhusen et al., 2020), such as higher levels of interleukin 6 (Breit et al., 2011; Wischhusen et al., 2020; Georgakis et al., 2021), and such inflammation can facilitate cholelithogenesis (Liu et al., 2018). GDF-15 can influence hepatic lipid metabolism and secretion and therefore alters biliary cholesterol secretion and bile concentration per se and *via* inflammation (Maurer et al., 2009; Luan et al., 2019). In addition, GDF-15 may be a proxy for immobilization (Conte et al., 2020), impaired fasting glucose (Hong et al., 2014), and insulin resistance (Vila et al., 2011; Kempf et al., 2012), which are risk factors for the development of gallstones.

Our study has several strengths, including MR design that strengthened causal inference, consistent associations in two independent populations, the confined study population of the European ancestry that minimized the population structure bias, stratification analysis by the diabetic status that diminished unobserved confounding from diabetes, and examination of nonlinearity. In addition, our study validated the use of GDF-15 as a potential therapeutic target to reduce the risk of diabetic complications which could be regarded as positive controls. Nevertheless, limitations need attention when interpreting our results. We observed pleiotropic associations for rs2517481 but not for other SNPs at the genome significance level. However, this pleiotropy was supposed to generate a limited impact on our findings since this SNP explains a small variance in GDF-15 levels, and the observed association between GDF-15 and gallstones was mainly driven by rs1227734 located in the *GDF15* gene region. The population confinement to European individuals may limit the generalizability of our findings to other populations, such as Asians and Africans. More research on GDF-15 in relation to gallstone disease in other populations is needed. In addition, the validity of genetic instruments for GDF-15 could not be examined in outcome data, given no phenotype information was available in UK Biobank and FinnGen. However, we obtained these SNPs from a hither to the largest genome-wide association meta-analysis study on GDF-15 (Folkersen et al., 2020), and the observed association appeared to be steered by the SNP located in the protein-encoding *GDF15* gene. Also, the confirmation of the shared causal variants between GDF-15 circulating levels and gallstone disease could not be examined due to insufficient data. Given that our findings are novel, a more functional study and randomized controlled trial (RCT) with causal inference potentials are needed to verify this association, and the corresponding underlying mechanisms also deserve further investigation.

In conclusion, this MR study suggests a detrimental effect of high GDF-15 levels on gallstone formation for the first time. This finding may indicate gallstone disease as a potential adverse outcome, when taking GDF-15 as a therapeutic target for diabetic complications as indicated by the metformin use.

## Data Availability

The original contributions presented in the study are included in the article/Supplementary Material; further inquiries can be directed to the corresponding author.
