# Supplementary material for: GDF-15 as a Therapeutic Target of Diabetic Complications Increases the Risk of Gallstone Disease: Mendelian Randomization and Polygenic Risk Score Analysis
Source: Front Genet. 2022 Jun 13;13:814457. doi: 10.3389/fgene.2022.814457 (PMC9234303; doi:10.3389/fgene.2022.814457)

Supporting information

**GDF-15 as a therapeutic target of diabetic complications increases the risk of gallstone disease: Mendelian randomization and polygenic risk score analysis**

*Lili Yu, Yajing Zhou, Lijuan Wang, Xuan Zhou, Jing Sun, Jiarui Xiao, Xiaolin Xu, Susanna C. Larsson,* *Shuai Yuan*, *Xue Li*

**Supplementary Table 1**. Phenotypes associated with genetic instruments for growth differentiation factor 15 at the genome-wide significance level

**Supplementary Figure 1.** Scatterplot of growth differentiation factor 15 associated genetic variants in relation to gallstones

**Supplementary Figure 2**. Leave-one-out analysis of growth differentiation factor 15 associated genetic variants in relation to gallstones

**Supplementary Table 1**. Phenotypes associated with genetic instruments for growth differentiation factor 15 at the genome-wide significance level

| **SNP** | **EA** | **Association** | **Direction** |
| --- | --- | --- | --- |
| rs2517481 | G | Different White blood cell count | - |
|  |  | Self-reported malabsorption or coeliac disease | + |
|  |  | Self-reported psoriasis | + |
|  |  | Primary sclerosing cholangitis | + |
|  |  | Height | + |
|  |  | Self-reported hypothyroidism or myxoedema | - |
|  |  | Rheumatoid arthritis | + |
|  |  | Intestinal malabsorption | + |
|  |  | Whole body fat-free mass | + |
|  |  | Basal metabolic rate | + |
|  |  | Weight | + |
|  |  | Unspecified haematuria | - |
|  |  | Hand grip strength | + |
| rs1227734 | T | Granulocyte percentage of myeloid white cells | + |
|  |  | Monocyte percentage of white cells | - |
| rs60164552 | G | Not available |  |
| rs112253475 | A | Not available |  |

EA, effect allele; SNP, single nucleotide polymorphism.

**Supplementary Figure 1.** Scatterplot of growth differentiation factor 15 associated genetic variants in relation to gallstones


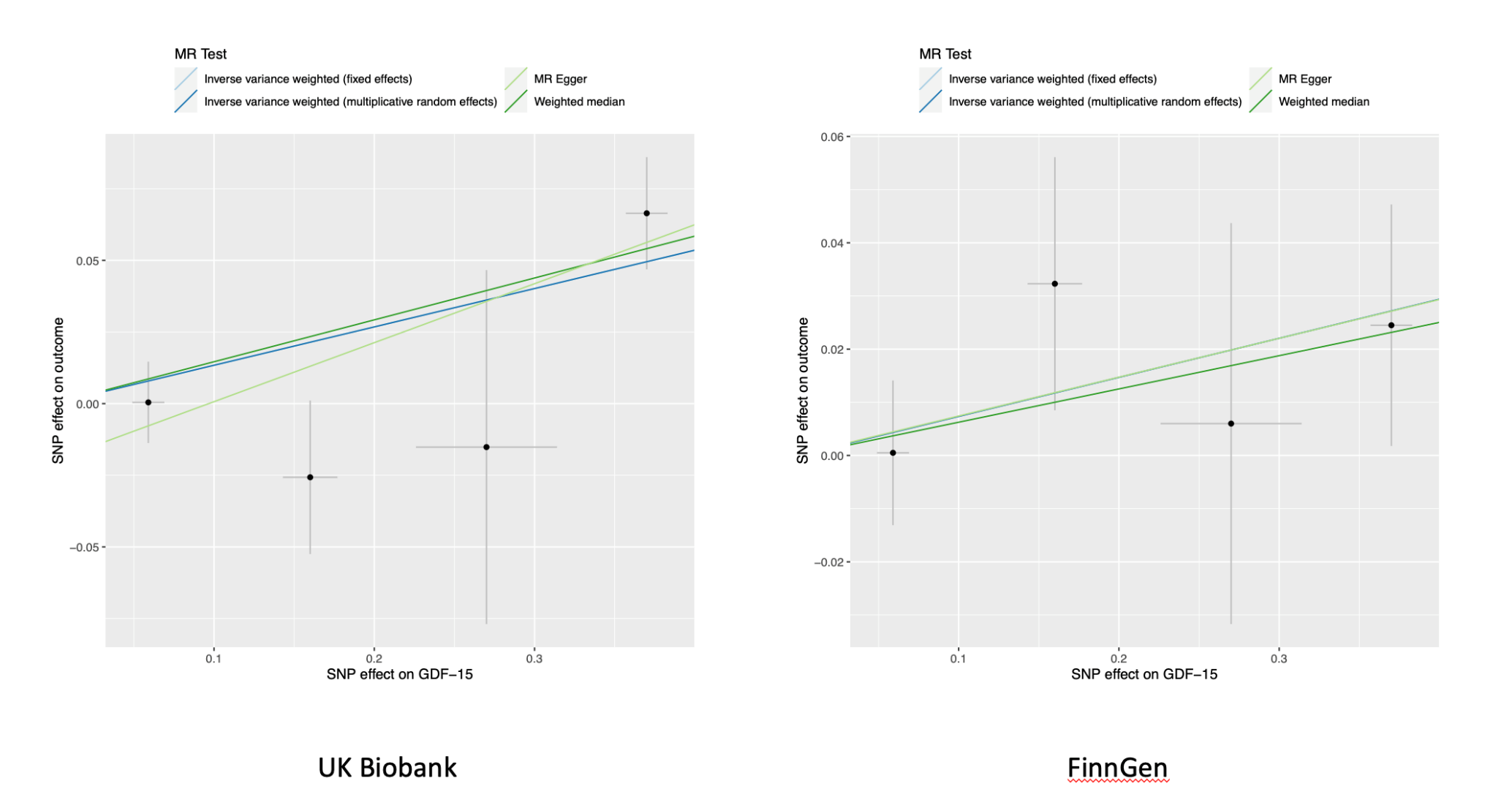


GDF-15, growth differentiation factor 15; MR, mendelian randomization; SNP, single nucleotide polymorphism.

**Supplementary Figure 2**. Leave-one-out analysis of growth differentiation factor 15 associated genetic variants in relation to gallstones


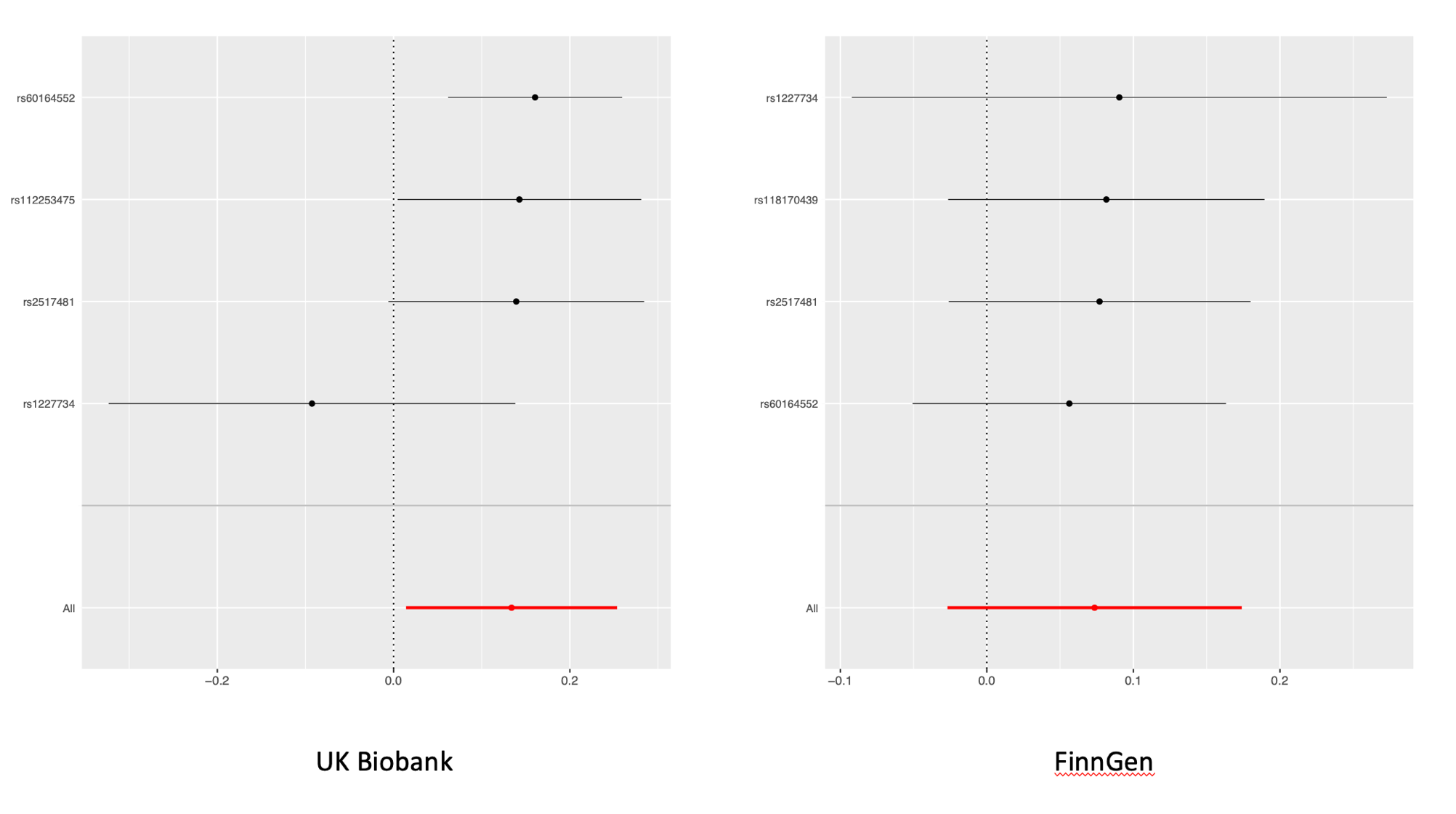

Supplement: Supplementary file 1 [file DataSheet1.docx]
